# Improving Black/African American Recruitment in ADRD Observational Studies: Black Women Inflammation and Tau Study

**DOI:** 10.1002/alz.091960

**Published:** 2025-01-09

**Authors:** Joy Stradford, Nadine Heyworth, Michelle Jackson, Marc Norman, April Thames, Sarah Banks, Erin E. Sundermann

**Affiliations:** ^1^ SDSU/ UCSD Joint Doctoral Program in Clinical Psychology, San Diego, CA USA; ^2^ University of California, San Diego, La Jolla, CA USA; ^3^ University of California, Los Angeles, Los Angeles, CA USA

## Abstract

**Background:**

Black/African American women are the most at‐risk demographic group for developing Alzheimer’s disease and related dementias (ADRD). Unfortunately, Black Americans are underrepresented in research, accounting for just 8% of clinical trial participants in the USA. Restrictive study exclusion criteria and medical mistrust are pervasive barriers to research participation in minoritized communities and are often disregarded during the development of a research study. The Black Women Inflammation and Tau Study (BWITS) is an observational study aiming to identify biopsychosocial risk factors for ADRD in Black women to improve health outcomes. Study development was informed by Community‐Based Participatory Research (CBPR) and National Institute on Minority Health and Health Disparities (NIMHD) Research Frameworks to address common participation challenges in minoritized groups.

**Method:**

BWITS is utilizing the CBPR and NIMHD frameworks to design the study and develop culturally informed and effective recruitment strategies. Recruitment is expected to begin in spring, 2024.

**Result:**

*Multidisciplinary Team*. The study has established a diverse multidisciplinary research team including Black neuropsychologists, neuroscientists, nurse practitioners, and scholars working together to address participants needs.

*Community Reciprocity & Partnerships*. BWITS strives to establish community reciprocity by providing education outreach to the community and communicating procedures, results, and providing health data to participants. The study team is actively working to build trust through community partnerships with Black community organizations and through routine consultation with a representative Community Advisory Board.

*Reducing Barriers*. BWITS will use minimally invasive blood draws to study ADRD biomarkers. To minimize time and transportation burden and increase accessibility, BWITS study visits will take place in convenient locations within predominantly Black communities of San Diego and Los Angeles with transportation options offered.

*Acknowledging Health Determinants in Minoritized Groups*. Diabetes affects 12.1% of Black American adults; however, individuals with diabetes are often ineligible from participating in ADRD studies resulting in disproportionate exclusion. BWITS has adopted broader eligibility criteria to combat this systemic problem.

**Conclusion:**

Understanding and incorporating the lived experiences of special populations should inform the foundation of any research study. The results of study enrollment will illustrate the significance of establishing a thoughtful recruitment plan for Black women research participants.

